# Incorporating Demand and Supply Constraints into Economic Evaluations in Low‐Income and Middle‐Income Countries

**DOI:** 10.1002/hec.3306

**Published:** 2016-01-19

**Authors:** Anna Vassall, Lindsay Mangham‐Jefferies, Gabriela B. Gomez, Catherine Pitt, Nicola Foster

**Affiliations:** ^1^ Department of Global Health and DevelopmentLondon School of Hygiene and Tropical MedicineLondonUK; ^2^ Department of Global Health, Academic Medical CenterUniversity of AmsterdamAmsterdamThe Netherlands; ^3^ Amsterdam Institute for Global Health and DevelopmentAmsterdamThe Netherlands; ^4^ Health Economics Unit, School of Public Health and Family MedicineUniversity of Cape TownSouth Africa

**Keywords:** economic evaluation, health system, demand, health technology assessment

## Abstract

Global guidelines for new technologies are based on cost and efficacy data from a limited number of trial locations. Country‐level decision makers need to consider whether cost‐effectiveness analysis used to inform global guidelines are sufficient for their situation or whether to use models that adjust cost‐effectiveness results taking into account setting‐specific epidemiological and cost heterogeneity. However, demand and supply constraints will also impact cost‐effectiveness by influencing the standard of care and the use and implementation of any new technology. These constraints may also vary substantially by setting. We present two case studies of economic evaluations of the introduction of new diagnostics for malaria and tuberculosis control. These case studies are used to analyse how the scope of economic evaluations of each technology expanded to account for and then address demand and supply constraints over time. We use these case studies to inform a conceptual framework that can be used to explore the characteristics of intervention complexity and the influence of demand and supply constraints. Finally, we describe a number of feasible steps that researchers who wish to apply our framework in cost‐effectiveness analyses.

## 1 Introduction

As new health technologies are developed, the World Health Organisation (WHO; 2012) considers whether to revise its guideline recommendations. Evidence on efficacy, cost and, in some cases, cost‐effectiveness may inform these revisions. Country‐level decision makers need to consider whether to adopt revised guidelines and fund the introduction of new technologies (Nasser *et al.*, 2014). Countries may consider the results of economic evaluations performed to inform global guidelines. However, unlike efficacy, cost‐effectiveness analyses are not necessarily generalisable beyond a specific setting (Drummond *et al.*, 2005). In theory, each country could conduct its own economic evaluation. However, in practice, such country‐specific analyses may be considered too time‐consuming and expensive for the majority of low‐income and middle‐income countries (LMICs). To support the rapid scale‐up of new technologies, global health actors have therefore invested in developing user‐friendly models that enable decision makers in LMICs to assess the cost‐effectiveness of introducing a given new technology in their own setting (Lubell *et al.*, 2008a; Winfrey *et al*., 2011; Pretorius *et al.*, 2014a; Pretorius *et al.*, 2014b).

Multi‐country cost‐effectiveness models typically apply efficacy data from trials and adjust for heterogeneity in demography, epidemiology, and unit costs (Lubell *et al.*, 2008a; Winfrey *et al.*, 2011; Pretorius *et al.*, 2014a; Pretorius *et al*., 2014b). Unit costs are adjusted either through cost models (using an ingredients approach and local input prices) or by using statistical models to extrapolate costs from empirical studies conducted in other settings. Cost and efficacy data are commonly entered as uncorrelated parameters. It is, however, rare for these models to explicitly consider other setting‐specific influences on cost‐effectiveness in LMICs, despite the fact that literature from high‐income countries (HICs) identifies a wide range of factors that are important to consider when transferring cost‐effectiveness estimates between settings (Drummond *et al.*, 2009). In particular, the use and therefore cost‐effectiveness of new technologies may be adversely influenced by constraints to the demand for and supply of health services in which the technology is placed.

This paper aims to encourage the consideration and incorporation of supply and demand constraints in economic evaluations of new health technologies in LMICs. Our objectives are threefold: first, to highlight the importance of considering constraints; second, to conceptualise constraints; and third, to propose pragmatic options for incorporating these constraints in economic evaluations. We begin by presenting a review of economic evaluations of new diagnostics for malaria and tuberculosis (TB) control and describe the extent to which these economic evaluations have considered demand and supply constraints and how these constraints influenced results. These two case studies are then used to develop a conceptual framework for identifying demand and supply constraints. Finally, we discuss the options and challenges in applying this framework going forward.

## 2 Economic Evaluations of Rapid Diagostic Tests for Malaria and Tuberculosis

In this section, we describe how the scope of economic evaluations conducted on new diagnostics for malaria and TB control evolved to incorporate demand and supply constraints. For this review, we broadly define ‘demand constraints’ as factors that restrict the individual's ability to seek and use healthcare services in a way that maximises the individual's utility. ‘Supply constraints’ are defined as factors that restrict the provider's ability to provide these services efficiently and with sufficient coverage and quality to have an optimal impact. We examine constraints that are additional to a payer's budget constraints.

In the case of malaria, we examine rapid diagnostic tests (mRDTs) at point of care. Prior to the rollout of mRDTs, most individuals with suspected malaria had limited access to facilities with microscopy (the previous standard of care) and were treated presumptively. Deploying mRDTs has the potential to reduce the overprovision of expensive anti‐malarials and to improve the clinical management of non‐malaria febrile illnesses. In the case of TB, we examine the implementation of Xpert MTB/RIF (hereafter referred to as Xpert), a test with improved sensitivity compared with microscopy (the previous standard of care) particularly for those co‐infected with HIV and TB (Boehme *et al.*, 2011). Xpert is also able to identify potential cases of multi‐drug‐resistant TB (MDR‐TB).

To identify economic evaluations on mRDTs, we searched PubMed, Scopus and the NHS Economic Evaluation Database for the terms ‘malaria’ and ‘cost’ and ‘rapid diagnostic’. Citations and bibliographies of studies retrieved and WHO malaria guidelines were reviewed for additional articles published between January 2004 and September 2015. For Xpert, we used two systematic reviews, one of modelling studies of novel diagnostic strategies for TB (Zwerling *et al.,* 2014) and one of TB control costs (Laurence *et al.,* 2015) and then updated them through a PubMed search for the terms ‘tuberculosis’ and ‘cost’ and ‘Xpert’. Citations and bibliographies of studies retrieved were reviewed for additional articles published up until September 2015.

We identified 27 economic evaluations on mRDTs and 10 economic evaluations on Xpert MTB/RIF (Table 1). We categorise them into three groups, according to the way in which they consider demand and supply constraints:

- *No adjustment.* Economic evaluations that include attributes of the new technology (such as test accuracy/efficacy), setting‐specific unit costs, and/or epidemiological considerations (such as disease prevalence) only.
- *Adjust for constraints.* Economic evaluations that include parameters listed in group A and also explore the influence of setting‐specific demand and supply constraints (such as care‐seeking preferences, patient adherence to treatment and/or provider practice).
- *Address constraints.* Economic evaluations that include parameters listed in groups A and B and also consider interventions to address demand and/or supply constraints (such as provider training, patient education and/or incentives).

- *Economic evaluations that do not adjust for demand and supply constraints*

**Table 1:** List of studies reviewed for the case studies on TB and malaria diagnostics

Disease	Reference	Scope	Analytical approach (perspective)	Countries	Comparators and interventions (including any critical enablers mentioned)	Main findings, and any observations on demand and supply side constraints (where no constraints mentioned, none considered)
Tuberculosis	(Vassall *et al*., 2011)	A	Decision analytic model (provider perspective)	India, South Africa, Uganda	(1) Microscopy	Xpert cost‐effective when compared with microscopy and clinical diagnosis, although dependent on current TB diagnostic practices, proportion of HIV‐TB co‐infection and prevalence of TB in population.
					(2) Xpert	
Tuberculosis	(Theron *et al*., 2012)	A	Decision analytic model (provider perspective)	South Africa	(1) Microscopy versus X‐ray (CXR)	If feasible, triaging using microscopy first, then followed by Xpert had the lowest cost per person diagnosed.
					(2) Latent TB testing (IGRA)	
					(3) Xpert	
Tuberculosis	(Abimbola *et al*., 2012)	A	Decision‐analytic model (provider perspective)	Sub‐Saharan Africa	(1) Microscopy and CXR	Assumed that persons with TB in the model started HIV treatment, and all TB positive received TB treatment immediately. Found that the ICER per death averted depended most strongly on the mortality among persons with undiagnosed TB.
					(2) Culture	
					(3) Xpert	
Tuberculosis	(Andrews *et al*., 2012)	A	Microsimulation mathematical model (provider perspective)	South Africa	(1) Microscopy	Screening all individuals initiating HIV treatment in South Africa with two Xpert samples is cost‐effective. Found strategies involving one Xpert sample or symptom screening less efficient.
					(2) Microscopy and culture	
					(3) one Xpert	
					(4) two Xperts	
Tuberculosis	(Menzies *et al*., 2012)	A	Transmission model (provider perspective)	Botswana, Lesotho, Namibia, South Africa, Swaziland	(1) Microscopy	Predicted substantial increases in HIV treatment costs following implementation of Xpert, which has a large influence on the cost‐effectiveness of Xpert. Concluded that Xpert was cost‐effective when compared against smear but was cautious in interpretation given uncertainty. Found that population transmission benefits of early detection were small.
					(2) Xpert	
Tuberculosis	(Meyer‐Rath *et al.,* 2012)	B	Decision‐analytic model (provider perspective)	South Africa	(1) Microscopy	Found that Xpert will increase number of cases diagnosed, number treated and cost of diagnosis. Also predicted that the total cost of treatment would double unless the inpatient model of MDR‐TB treatment is replaced with an outpatient model.
					(2) Xpert (with sub‐studies examining Xpert placement)	
Tuberculosis	(van't Hoog *et al.,* 2013)	B	Decision‐analytic model (provider perspective)	Uganda, India, South Africa	(1) Xpert	A triage test (such as X‐ray) to target Xpert reduced diagnostic costs by 42% in Uganda, 34% in India and 39% in South Africa. Triage tests with lower sensitivity resulted in a similar cost reduction but were not cost‐effective relative to the WHO threshold in India and South Africa.
					(2) Xpert with triaging	
Tuberculosis	(Langley *et al.,* 2014)	B	Transmission model combined with a health system/operational model (provider perspective)	Tanzania	(1) Microscopy	Found that Xpert would be the most cost‐effective strategy, using an operational model parameterised with local health system and cost data
					(2) Xpert	
Tuberculosis	(Foster *et al.*, 2015)	C	Pragmatic trial informed decision‐analytical model (societal perspective)	South Africa	(1) Microscopy	Cost‐effectiveness analysis based on model using data from pragmatic trial (Churchyard *et al.*, 2015) that finds Xpert MTB/RIF to have no impact on mortality. This analysis compares Xpert only introduction to introduction with investments in the TB case pathway and finds that while Xpert alone is unlikely to be cost‐effective, it will be with supporting activities
					(2) Xpert	
					(3) Microscopy with supply‐side strengthening	
					(4) Xpert with supply‐side strengthening	
					(5) Xpert	
Tuberculosis	(Menzies *et al.*, 2015)	B	Transmission model (provider perspective)	Botswana, Lesotho, Namibia, South Africa, Swaziland	(1) Microscopy	Revisitng of 2011 paper by same authors to adjust results for different assumptions about presumptive treatment and finds a 60% increase in the incremental cost‐effectiveness ratios for Xpert adoption.
					(2) Xpert	
Malaria	(Bualombai *et al.*, 2003)	A	Clinical trial‐based (provider and patient perspective)	Thailand	(1) Microscopy	RDT (OptiMal) was the most cost‐effective and policy makers should consider using RDT technology to supplement microscopy in remote non‐microscope areas.
					(2) RDT (Optimal)	
					(3) RDT (Immuno‐chromatographic test)	
Malaria	(Rolland *et al.*, 2006)	A	Decision‐analytic model (provider perspective)	Sub‐Saharan Africa	(1) Presumptive diagnosis	Cost‐effectiveness of RDT depends on malaria prevalence, cost of RDT and cost of treatment (with artesunate plus amodiaquine or artemether‐lumefantrine). In most epidemic prevalence scenarios, RDTs would considerably reduce over‐treatment for only a moderate increase in costs over presumptive diagnosis. A substantial decrease in RDT unit price would greatly increase their cost‐effectiveness and should thus be advocated.
					(2) RDT	
Malaria	(Lubell *et al.*, 2007)	B	Decision‐analytic model (provider perspective)	Tanzania	(1) Microscopy	If prescribers comply with current guidelines, microscopy would give rise to lower average costs per patient correctly treated than RDTs in areas of both high and low transmission.
					(2) RDT	Cost‐effectiveness would be worse if prescribers do not comply with test results. The cost of this additional benefit may be higher than many countries can afford without external assistance or lower RDT prices.
Malaria	(Lubell *et al.*, 2008a)	B	Decision‐analytic model (illustrative example using trial data)	Uganda	(1) Presumptive diagnosis,	An interactive model designed to assist policy makers. Results show cost‐effectiveness depends on location and is sensitive to malaria transmission intensity, costs, and accuracy of RDT, and provider adherence to negative test results. Once provider adherence to negative test results fell below 65% presumptive treatment became the preferred option.
					(2) RDT (two types)	
Malaria	(Lubell *et al.*, 2008b)	B	Decision‐analytic model using on clinical trial data (societal perspective)	Tanzania	(1) Presumptive diagnosis	Improving diagnostic methods, including RDTs, can reduce costs and enhance the benefits of effective anti‐malarial drugs, but only if the consistency of response to test results is also improved. Investing in methods to improve rational response to tests is essential. Economic evaluations of diagnostic tests should take into account whether clinicians' response is consistent with test results.
					(2) Microscopy	
					(3) RDT	
Malaria	(Shillcutt *et al.*, 2008)	A	Decision‐analytic model (societal perspective)	Sub‐Saharan Africa	(1) Presumptive diagnosis	RDTs were cost‐effective compared with presumptive diagnosis in most settings and probably cost‐effective compared with microscopy, reflecting better accuracy in improved conditions. CE mainly reflects improved treatment and health outcomes of non‐malaria febrile illness plus savings in anti‐malarial drug costs. Results depend on assumption that providers use test results in treatment decisions.
					(2) Microscopy	
					(3) RDT	
Malaria	(Zikusooka *et al.*, 2008)	A	Clinical trial‐based (provider perspective)	Mozambique	(1) Presumptive diagnosis	RDTs are cost‐saving when malaria prevalence is low to medium. Compared with treating patients on the basis of presumptive diagnosis, the use of RDTs results in cost savings only when 29% and 52% or less of all suspected malaria cases test positive for malaria and are treated with artesunate plus sulfadoxine/pyrimethamine (AS + SP) and artemether‐lumefantrine (AL), respectively. These cut‐off points increase to 41.5% (for AS + SP) and to 74% (for AL) when the use of RDTs is restricted to only those older than 6 years of age.
					(2) RDT	
Malaria	(Zurovac *et al.*, 2008)	B	Decision‐analytic model using data on actual clinical practice (provider perspective)	Kenya	(1) Presumptive diagnosis	In the high transmission district, RDTs as actually used would improve malaria treatment and lower costs, but the majority of patients with malaria would not be correctly treated. In the low transmission district, RDTs as actually used would yield a minor reduction in under‐treatment errors with 41% higher costs. In both districts, adherence to revised clinical practices with RDTs has the potential to further decrease treatment errors with acceptable costs.
					(2) RDT	
Malaria	(Chanda *et al.*, 2009)	B	Clinical trial‐based (provider perspective)	Zambia	(1) Presumptive diagnosis	RDTs were the most cost‐effective method at correctly diagnosing malaria in primary health facilities when compared with presumptive diagnosis and microscopy. Prescribing practices can impact on the potential of RDTs to achieve overall cost savings.
					(2) Microscopy	
					(3) RDT	
Malaria	(Rosas Aguirre *et al.*, 2009)	A	Decision‐analytic model (provider perspective)	Peru	(1) Presumptive diagnosis	RDTs were cost‐effective compared with presumptive diagnosis and microscopy
					(2) Microscopy,	
					(3) RDT	
Malaria	(Uzochukwu *et al.*, 2009).	A	Decision‐analytic model (societal perspectives)	Nigeria	(1) Presumptive diagnosis	RDT is cost‐effective compared with presumptive diagnosis at malaria prevalence of 43% and therefore suitable in Nigeria. Cost‐effectiveness was affected by malaria prevalence level, ACT adherence level, cost of ACT, proportion of non‐malaria febrile illness cases that were bacterial, and microscopy and RDT sensitivity.
					(2) RDT	
Malaria	(Faye *et al.*, 2010)	A	Trial‐based (provider perspective)	Senegal	(1) Presumptive diagnosis	RDT was cost‐effective compared with presumptive diagnosis
					(2) RDT	
Malaria	(de Oliveira *et al.*, 2010)	A	Decision‐analytic model (provider perspective)	Brazil	(1) Microscopy	Microscopy was more cost‐effective than RDT in remote areas of Brazil if high accuracy of microscopy is maintained in the field. Decision regarding use of RDTs in these remote areas depends on the current microscopy accuracy in the field.
					(2) RDT	
Malaria	(Ly *et al.*, 2010)	A	Decision‐analytic model based on cohort study data (provider perspective)	Senegal	(1) Presumptive treatment of all fevers	RDT use for all clinically suspected malaria and prescribing ACT only to patients tested positive was cost‐effective in areas where microscopy is unavailable. Full compliance of providers with RDT results was required in order to avoid severe incremental costs.
					(2) Presumptive treatment according to clinician's judgement	
					(3) Treat all cases of illness that are RDT‐positive only (the national policy)	
					(4) Treat all fevers that are RDT‐positive only	
					(5) Treat all children under 6 years presumptively and others with fever and positive RDT (WHO guidelines)	
Malaria	(Yukich *et al.*, 2010)	A	Trial‐based (societal perspective)	Tanzania	(1) Microscopy	RDTs reduced drug costs in this setting but did not offset the cost of the tests. Non‐monetary benefits included improved management of patients and increased compliance with test results.
					(2) RDT	
Malaria	(Batwala *et al.*, 2011)	A	Decision‐analytic model based on trial data (societal perspective)	Uganda	(1) Presumptive diagnosis	RDT was more cost‐effective than microscopy in both low and high transmission settings and considered cost‐effective with respect to presumptive treatment.
					(2) Microscopy,	
					(3) RDT	
Malaria	(Chanda *et al.*, 2011)	C	Trial‐based (provider perspective)	Zambia	(1) Facility (RDT and treatment)	Home management of uncomplicated malaria using community health workers was more cost‐effective than facility‐based management. Utilisation and adherence to clinical guidelines was higher with community health workers than at a health facility.
					(2) Home management using community health workers (RDT and treatment)	
Malaria	(Lemma *et al.*, 2011)	A	Clinical trial‐based (provider perspective)	Ethiopia	(1) Presumptive diagnosis	RDT (pan/pf) was more cost‐effective than both RDT (pf) and presumptive disgnosis and should be preferred in health posts in rural Tigray and rolled out nationwide.
					(2) RDT (pf/pan),	
					(3) RDT (pf)	
Malaria	(Thiam *et al.*, 2011)	B	Trial‐based (provider perspective)	Senegal	(1) Presumptive diagnosis	RDT was cost‐effective compared with presumptive diagnosis, but highlight impact of drug stock outs, and improved adherence to RDT results over time.
					(2) RDT	
Malaria	(de Oliveira *et al.*, 2012)	A	Decision‐analytic model (provider perspective)	Brazil	(1) Microscopy	Microscopy is more cost‐effective than RDT in the remote areas studied if high accuracy of microscopy is maintained in the field.
					(2) RDT	
Malaria	(Ansah *et al.*, 2013).	B	Decision analytic model based on clinical trial (provider perspective)	Ghana	(1) Presumptive diagnosis	Compared with a presumptive diagnosis, RDTs increased the proportion of patients who were correctly treated in relation to treatment with anti‐malarials, from 42% to 65% at an incremental societal cost of US$8.3 per additional correctly treated patients. In the ‘microscopy setting’, there was no advantage to replacing microscopy by RDT as the cost and proportion of correctly treated patients were similar. Results were sensitive to the cost of RDTs and to improvements in adherence to negative tests.
					(2) Microscopy	
					(3) RDT	
Malaria	(Bisoffi *et al.*, 2011).	A	Decision‐analytic model based on trial data (provider perspective)	Burkina Faso	(1) Presumptive diagnosis	A febrile child under 5 years should be treated presumptively. In the dry season, the probability of clinical malaria in adults is so low, that neither testing nor treating with any regimen should be recommended. In the rainy season, if costs are considered, a febrile adult should not be tested, nor treated with ACT, but a possible alternative would be a presumptive treatment with amodiaquine plus sulfadoxine‐pyrimethamine. If costs were not considered, testing would be recommended.
					(2) RDT	
Malaria	(Harchut *et al.*, 2013)	B	Trial‐based (provider perspective)	Tanzania	(1) Microscopy	A marked difference between the number of positive malaria cases diagnosed with microscopy and RDT suggests that malaria is being over‐diagnosed by 64% with microscopy in this rural region of Tanzania. RDTs were cost‐effective compared with microscopy.
					(2) RDT	
Malaria	(Basu *et al.*, 2014)	B	Transmission model (societal perspective)	Six countries in sub‐Saharan Africa	(1) Presumptive diagnosis	A threshold transmission rate exists under which malaria testing is more cost‐effective than presumptive diagnosis and treatment.
					(2) RDT	
Malaria	(Mangham‐Jefferies *et al.*, 2014)	C	Trial‐based with individual patient‐level analysis (societal perspective)	Cameroon	(1) Microscopy	Introducing RDTs with enhanced training was more cost‐effective than with basic training when each was compared with microscopy.
					(2) RDT with basic training	
					(3) RDT with enhanced training	
Malaria	(Chen *et al.*, 2015)	C	Decision‐analytic model (provider and patient perspective)	Myanmar	(1) RDT but no supporting intervention	Private provider subsidies with IEC or a combination of IEC and financial incentives may be a good investment for malaria control.
					(2) RDT subsidy	
					(3) RDT subsidy with financial incentives	
					(4) RDT subsidy with information, education and counselling (IEC)	
Malaria	(Hansen *et al.*, 2015)	B	Decision‐analytic model based on trial data (provider perspective)	Afghanistan	(1) Presumptive diagnosis	RDTs were cost‐effective compared with microscopy and presumptive diagnosis across the moderate and low transmission settings. RDT remained cost‐effective even when microscopy was used for other clinical purposes.
					(2) Microscopy	
					(3) RDT	

Where the Scope of Economic evaluation is defined as (A) Economic evaluations that include attributes of the new technology, setting‐specific unit costs and epidemiological considerations; (B) Economic evaluations that include parameters listed in (A) and also potential demand and supply constraints; (C) Economic evaluations that include parameters listed in type B, and the intervention includes activities that address demand and supply constraints.

Half (14 of 27) of the economic evaluations conducted on mRDT did not adjust for any demand or supply constraints (Table 1). Diagnosis using mRDTs is compared with presumptive diagnosis, microscopy or both, and the studies include parameters on the accuracy of the alternative diagnostic methods, combined with setting‐specific data on unit costs of diagnosis and treatment and the prevalence of malaria. The cost‐effectiveness of mRDTs and microscopy varied across the studies and was influenced by setting‐specific unit costs and prevalence. While demand and supply constraints were not investigated directly, some studies noted that their findings were dependent on assumptions made regarding the ‘field’ accuracy of microscopy, prescribers' use of test results and patients' adherence to treatment.

In the case of Xpert, four of the 10 economic evaluations did not explicitly consider supply or demand constraints. In a similar manner to the studies on mRDTs, these studies considered unit costs and difference in epidemiology by setting. While there was no explicit consideration of any constraints, some studies included costs for training and quality control of Xpert (Vassall *et al.,* 2011). Sensitivity analyses were also conducted around the performance of other tests in the diagnostic pathway and the level of HIV care coverage, but no constraints on either provider or patient adherence to the TB diagnostic or the treatment pathway were considered (Abimbola *et al.*, 2012; Andrews *et al.*, 2012; Menzies *et al.*, 2012).

*Economic evaluations that adjust for potential demand and supply constraints*

Ten of the economic evaluations conducted on mRDTs explicitly adjusted for demand and supply constraints. One of the first studies explored how provider behaviour can impact on the cost‐effectiveness of mRDTs using trial data from Tanzania (Lubell *et al.*, 2008b). The authors concluded that neither mRDTs nor microscopy would be beneficial given the high rate at which anti‐malarials were prescribed to patients who tested negative for malaria. This study found that costs incurred would be 10–250% higher (depending on transmission rate) than if prescribing practice were fully consistent with test results. Similar findings were reported in Kenya, where an economic model populated with observed data on provider practice found that introducing mRDTs in a low transmission area would incur 41% higher costs and achieve only a small reduction in under‐treatment errors than the standard of care (Zurovac *et al.*, 2008). Other studies investigated the sensitivity of findings to the locally achieved accuracy of microscopy (Harchut *et al.*, 2013).

In the case of Xpert, this category contains two sub‐categories of studies. In the first set are three studies, which considered how the organisation of health services may affect the cost‐effectiveness of Xpert. These studies explored how the placement of Xpert in sites with different workloads influenced costs (Meyer‐Rath *et al.*, 2011), the availability and use of other diagnostic tests (van't Hoog *et al.*, 2013) and capacity constraints at the facility and district level (Lin *et al.,* 2011). The second category of studies evaluate the cost‐effectiveness of Xpert during scale‐up in ‘early adopter’ countries using large‐scale pragmatic trials. Preliminary results from these studies suggested that effective presumptive TB treatment, lack of access to HIV treatment, high levels of default from the diagnostic process and poor adherence by both clinicians and patients to guidelines following negative test results may substantially reduce the cost‐effectiveness of Xpert (Theron *et al.*, 2014a; Theron *et al.*, 2014b; Churchyard *et al.*, 2015). When the results of these trials were taken into consideration, estimates of incremental cost‐effectiveness ratios (ICERs) for a range of settings increased by up to 60% (Menzies *et al.,* 2015).

*Economic evaluations that address demand and supply constraints*

Three of the economic evaluations on mRDTs extended the scope of the ‘mRDT’ intervention to include activities to address demand and supply constraints. For example, in Cameroon, an economic evaluation assessed the introduction of mRDTs with two types of provider training: basic training on revised malaria guidelines and an enhanced training, which supplied information on the guidelines and targeted provider behaviour (Mangham‐Jefferies *et al.*, 2014). The study showed that introducing mRDTs with enhanced training was more expensive than mRDTs with basic training, but also more cost‐effective. In Myanmar, researchers evaluated four strategies for introducing mRDTs and concluded that introducing subsidised RDTs in the private sector accompanied by information, education and communication (IEC) and/or a combination of IEC and financial incentives would be cost‐effective (Chen *et al.*, 2015).

Only one Xpert study has analysed the cost‐effectiveness of activities to address supply or demand constraints. This study examined the combined cost‐effectiveness of Xpert with activities to support adherence to the diagnostic algorithm (follow‐up of those defaulting, follow‐up of negative test results in those with HIV and linkage to HIV care) in South Africa and found much higher ICERs than originally predicted (Foster *et al.*, 2015).

In summary, our case studies suggest that failing to consider demand and supply constraints may overestimate cost‐effectiveness. Although these case studies do not represent the vast array of new technologies in all LMICs, they are particularly important cases given the substantial investments being made in rapid diagnostics. The inclusion of constraints in both cases substantially influenced estimates of ICERs, and this suggests that constraints should be routinely considered in economic evaluations, at least to the same extent as heterogeneity in epidemiology is considered across settings. It could be argued that evaluating new technologies in the context of demand and supply constraints may ‘unfairly’ penalise new technologies for factors that are outside their control. However, including the cost of activities that address constraints (or reducing the estimated effect in their absence) rewards more feasible technologies, which is the primary innovation for many new technologies designed for use in LMIC's. As a decision and policy‐oriented field, economic evaluation is concerned with minimising the actual opportunity cost in the decision makers' jurisdiction, rather than a hypothetical one.

## 3 Conceptual Framework

Informed by the case studies, we consider two approaches to incorporate demand and supply constraints in economic evaluations. The first is to view the technology and activities to address constraints (sometimes referred to as ‘critical enablers’ (Schwartlander *et al.*, 2011)) as distinct interventions and to assess their cost‐effectiveness separately. This approach is analogous to those distinguishing between the efficacy of a technology and its effectiveness during implementation in the clinical literature and underpins the economic evaluation approach taken in our case studies (first evaluate based on efficacy, then examine the incremental cost‐effectiveness of supporting activities).

Alternatively, we propose that researchers should start from the perspective that new technologies are not ‘interventions’ in themselves. Many new technologies will require some supporting activity to achieve satisfactory efficacy and coverage objectives, given the extensive demand and supply side constraints in LMICs. We recommend that researchers should therefore routinely assume that the introduction of new technologies is likely to be an inherently complex intervention with many component activities. There is a substantive literature on how to determine whether an intervention is complex (Hawe *et al.,* 2004; Shiell *et al.,* 2008). In general, complex interventions are characterised as having several interacting activities and typically require those delivering or receiving the intervention to modify their behaviour. We accept that not all new technologies will fulfil these criteria. The extent to which introducing a new technology requires a complex intervention may depend on the nature of the technology, the extent of constraints, and the scale of the technology required. However, we propose that, given the extent of constraints, complexity needs to be ‘ruled out’ rather than ‘ruled in’ in economic evaluations in LMICs.

To assist in this process, we outline in the succeeding discussions a framework for researchers firstly to define the intervention they are evaluating and thereafter to guide their economic evaluation design. Some argue that while complexity may raise additional questions, with adequate time and resources, existing economic evaluation frameworks remain appropriate (Shiell *et al*., 2008). However, in the case of economic evaluation in LMICs, the extensive task of characterising, identifying and modelling intervention complexity remains a barrier for widespread global application.

Previous work on transferability frameworks from high‐income country (HIC) settings identifies a range of factors that influence economic evaluation results by setting, including patient, clinician, healthcare system and wider socio‐economic factors (Sculpher *et al.*, 2004; Drummond *et al.*, 2005). The ISPOR Good Research Practice Task Force report on the ‘Transferability of Economic Evaluations across Jurisdictions’ also identifies similar considerations (Drummond *et al.*, 2009). Many of these setting‐specific determinants of cost‐effectiveness fall within our definition of demand and supply constraints in the context of LMICs. There is also a body of literature from health systems research assessing the relationships between various new technologies, the health system and patient outcomes (Atun *et al.*, 2010a; Atun *et al.*, 2010b); a number of frameworks such as the WHO's ‘Building Blocks’ that provide insight into the range of supply constraints; and conceptual frameworks examining demand‐side constraints for health services (Levesque *et al.*, 2013).

**Figure 1:**
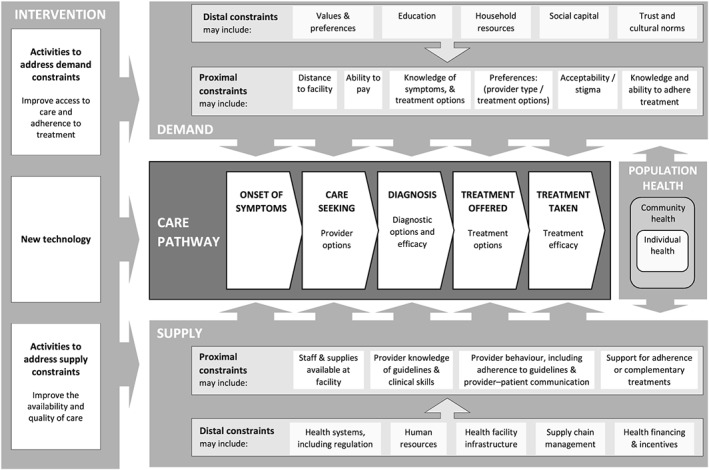
Conceptual framework for identifying the intervention and constraints in the context of the disease/illness/care pathway

Building on this work, in Figure 1, we present a visual conceptual framework to help researchers identify demand and supply constraints. This conceptualisation promotes a comprehensive description of the intervention, including the technology and activities to support its optimal use. The disease/illness/care pathway lies at the centre of the conceptual framework. We depict a simple disease/illness/care pathway that starts from onset of symptoms and continues to care seeking, diagnosis, treatment offered and treatment taken. This form of pathway needs to be specified in sufficient detail to include both disease progression and key decisions taken to seek/provide care. Additional stages could be added, for example, if the diagnostic process involved multiple sequential tests.

Having specified the pathway, including key decision points and options at each stage, the next step is to consider (i) how key decision points to seek/provide care are influenced by the new technology, (ii) the extent to which decisions/options are likely to be constrained by demand and supply factors, and (iii) what actions are needed to address these demand or supply constraints. Specifying the theory of change (De Silva *et al.*, 2014) outlining how the new technology is expected to impact the pathway and outcomes may help in this task.

Some constraints on decision points in the pathway may only become apparent as new technologies are scaled up and may go beyond a direct influence on the technology. For example, while suboptimal provider behaviour may be noticeable at low coverage, more general, human resource constraints may only become apparent at scale. Likewise, on the demand side, uptake of a new technology may be relatively straightforward for users who are frequent health service attendees, but there may be additional costs of ensuring access to hard‐to‐reach groups at higher levels of scale. We have therefore sought to distinguish proximal and distal constraints in our framework. On the demand side, proximal constraints include those that directly restrict access, uptake or adherence to the optimal patient pathway. For instance, care seeking may depend on the ease and cost of accessing care, individuals' awareness of symptoms and treatment options and perceptions about the quality of care. These proximal constraints are in turn influenced by household economics and underlying values and preferences that are shaped by the economic, social and cultural context. On the supply side, proximal constraints may include the knowledge and behaviour of healthcare providers and the availability of complementary resources and services. However, these may be constrained by distal factors, such as overall human resource availability; mechanisms for healthcare financing, planning and management; and the functioning of private sector providers.

We demonstrate how the conceptual framework can be used by applying it to the introduction of mRDTs (Table 2). The first column of Table 2 depicts the different stages in the pathway, and the subsequent columns then consider (i) the key decisions (and options) faced by the patient and provider; (ii) how the new technology should change the pathway; (iii) which proximal demand and supply factors may constrain the use of the technology; and (iv) which interventions may be required to address the demand and supply constraints. For example, mRDTs should extend access to malaria testing and should reduce the number of febrile patients who unnecessarily receive anti‐malarials. However, these outcomes may be constrained by the knowledge and preferences of both patients and providers, by patients' ability to access and pay for care, and by whether mRDTs, anti‐malarials and treatments for non‐malaria febrile illnesses are in stock. Table 2 has been used in practice. Many of the demand and supply constraints listed here were identified when designing interventions to support the rollout of mRDTs in Cameroon and were underpinned by a theory of change evaluation and formative research (Achonduh *et al.*, 2014). The final column lists activities to address these demand and supply constraints. In Cameroon, the interventions focused on provider knowledge and behaviour (Mbacham *et al.*, 2014; Mangham‐Jefferies *et al.,* 2014), although elsewhere, activities have aimed to raise community awareness of mRDTs (Onwujekwe *et al.*, 2015) or subsidise the cost of RDTs (Chen *et al.*, 2015).

**Table 2:** Illustrative example: applying the conceptual framework to the introduction of rapid diagnostic tests for malaria

Stage In pathway	Key decisions and options	How does the new technology change the care pathway?	What are the proximal demand and supply constraints?	What interventions may be required to address demand and supply constraints?
Onset of symptoms	Fever is a symptom of malaria, but not all fevers are caused by malaria.			
	Prevalence of malaria depends on malaria transmission rate in a given locality			
Care seeking	Where do individuals with a fever seek treatment?	• mRDTs should extend access to malaria testing as mRDTs can be used without specialist facilities or staff.	• In a given locality, types of provider offering malaria treatment, and the ease and cost of access	• Health promotion on malaria testing to encourage use of facilities that offer testing
	Many different types of provider offer malaria treatment (including public health facilities, private clinics, pharmacies, drug stores, itinerant drug vendors and traditional healers)	• mRDTs may change care‐seeking behaviour (depends on patient preferences over testing)	• Patient awareness and preferences over different types of provider and services available	
Diagnosis	What are the current options for diagnosis? How accurate are they?	• mRDTs should extend access to malaria testing (may replace or complement existing methods)	• Availability of microscopy (requires laboratory, equipment and specialist staff) and mRDTs	• Clinical guidelines are revised and distributed
	• Symptomatic diagnosis: highly inaccurate, with many false positives because not all fevers are caused by malaria	• mRDT are highly accurate (although some argue microscopy remains the gold standard)	• Providers knowledge of malaria symptoms	• Provider training on how to conduct and interpret mRDT (and encouraged providers to test before treat)
	• Microscopy (available at some but not all types of provider): considered the gold standard, although studies have shown more false positives in ‘routine’ than ‘expert’ microscopy.	• mRDTs should mean more febrile patients are tested for malaria before treatment is prescribed	• Providers ability to conduct and interpret microscopy and mRDT	• Health promotion on malaria testing and mRDT
			• Patient and provider preferences over diagnostic methods (many prefer presumptive treatment)	• Subsidise cost of diagnostic testing
			• Patient ability to pay for malaria test (microscopy or mRDT)	
Treatment offered	What are the treatment options?	• mRDTs should reduce overtreatment as anti‐malarials are targeted to febrile patients with a confirmed diagnosis	• Provider knowledge of the clinical guidelines	• Provider training on revised clinical guidelines (and intervention to change prescribing preferences and reduce overtreatment)
	• Artemisinin combination therapy (ACT) is first‐line treatment		• Provider adherence to clinical guidelines (i.e. treat only test‐positive patients)	• Patient education to change expectations (not all fevers are malaria)
	• Other anti‐malarials, include sulfadoxine pyrimethamine (SP), amodiaquine and chloroquine		• Availability of prescribed treatment	• Improve anti‐malarial supply chain
			• Patient and provider preferences over alternative treatments	
Treatment taken	Are they efficacious?		• Patient ability to pay for treatment (ACT is more expensive than other anti‐malarials)	• Subsidise cost of treatment
	• ACT is highly efficacious		• Provider advice/patient knowledge on treatment regimen	• Provider intervention to improve treatment advice
	• Efficacy of other anti‐malarials is limited (malaria parasites are increasingly resistant)			• Patient education on treatment regimen

It should also be noted that we do not recommend that the process of developing Table 2 is one conducted by the economic evaluator alone. Defining the intervention to introduce a new technology should be a multi‐stakeholder process. It is feasible for economists to work with programme/technology experts to arrive at an outlined Table 2 before embarking on the model and cost‐effectiveness analysis design (Onwujekwe *et al.,* 2015). It should also be noted that the resulting intervention may not address all constraints. Some activities may still be considered by stakeholders as areas to address during implementation. Even in this case, our framework encourages the researcher to consider the full cost of those activities that are agreed and to understand where the case pathway should be constrained in their analysis, even if the implementation of activities to address constraints is postponed.

## 4 Applying the Conceptual Framework

When considering how to account for or address demand and supply constraints, the potential gains from avoiding sub‐optimal investment decisions must be weighed against the risk of delaying introduction of technologies with high benefits by waiting for new evidence to emerge from more complex economic analyses. While it may be feasible to work through the framework a priori, the data required to explore such a framework empirically are potentially immense. We identify in the succeeding discussions several approaches to expedite and support the application of this framework in different analytical situations.

### 4.1 Economic evaluations alongside early trials

Work on transferability from HICs identifies various options for using trial data in economic evaluations either when individual patient‐level data are available or when the trial took place in the country of interest (Manca and Willan, 2006). In the majority of cases of new technology adoption in LMICs, however, neither of these two situations exists. Nevertheless, early trials of new technologies may still offer some opportunities to inform economic evaluations incorporating constraints. Many trials of new technologies in LMICs are multi‐centre and thus provide scope to identify and collect data on location‐specific constraints. However, opportunities may be limited as the research process masks a number of constraints that would be observed elsewhere. Some argue that trial sites could be selected to represent the range of settings into which the new technology may be placed, incorporating any prior knowledge about key demand and supply constraints to improve representativeness (Gheorghe *et al.*, 2014). However, given the shortage of ‘trial ready’ sites in LMICs, and practical issues such as distance and transportation to and from sites in LMICs, there may be a substantial trade‐off between speed and expense of trial start‐up and ensuring that sites are representative.

Despite these limitations, at the very least, detailed descriptive reporting of the cost to ensure the implementation of both the standard of care and the intervention in trials should become standard practice. This would not only help define the intervention but may also provide some information to estimate cost for other settings. This recommendation is supported by the literature on the methodological implications of evaluating complex interventions, which emphasises the need for detailed and standardised reporting about the nature of the intervention and the implementation process (Shiell *et al.*, 2008).

In HICs, considerable attention has been placed on the analysis of patient‐level data from trials to explore heterogeneity across locations and individuals (Grieve *et al.*, 2005; Gomes *et al*., 2012). Where constraints are likely to operate at a sub‐group or individual level, this additional data may add considerable insight. However, to date, there have been few trial‐based cost‐effectiveness analyses that report patient‐level data on costs and cost‐effectiveness from LMICs. This is likely to be due in part to the substantial costs of data collection involved, where routine systems for resource use are non‐existent. Nonetheless, work is beginning to emerge in this area (Barton *et al.*, 2013; Mangham‐Jefferies *et al.*, 2014), and this approach offers the promise of adding critical insight into a number of constraints, most notably, demand constraints that may operate at an individual level.

### 4.2 Adapting model‐based economic evaluations to consider supply and demand

Given the substantial limitations of trials, it is likely that for some time to come, analysts will continue to use both trial data and models to predict the cost‐effectiveness of new technologies in LMICs. In this case, there are several approaches that may be considered. The first option is perhaps the most intensive, viewing demand and supply constraints with the same importance and rigour as any other determinant of cost‐effectiveness incorporated in a model. This approach would involve applying our framework and conducting a systematic review on the influence of supply and demand constraints on key pathway probabilities and conducting a wide range of sensitivity analyses. This would serve the purpose of further defining which constraints are most important and could help focus activities to address these constraints.

However, the data scarcity around many of these constraints in LMICs means that populating models with plausible data may be problematic. For example, in our case studies, there was very little prior data available on the levels of presumptive treatment in TB (Theron *et al.*, 2014a). Even if the presumptive treatment rate is identified as a key determinant of cost‐effectiveness, it is hard to adjust for presumptive treatment rates in any particular location. It may, however, be feasible to collect additional data as part of model‐based evaluations, rather than solely rely on systematic reviews. For example, it may be possible to estimate the costs of activities to address constraints from other new technologies within the same country and conduct small observational studies in areas like clinician behaviour. Such studies may have the wider benefit of ensuring results resonate with decision makers, who may have priors from previous experiences regarding the ‘real’ costs of implementation in their setting.

Where the literature indicates a constraint may be important but no local data are available, we recommend at the very least that the uncertainty associated with the constraint be communicated with decision makers. There are several examples of this approach emerging in the literature. Recent work by Gomez *et al.* models shortened TB drugs regimens in a set of scenarios where full guideline adherence by healthcare providers and patients is compared with ‘real world’ scenarios (Gomez *et al.*, 2015). Another approach to explore constraints is ‘cascade’ modelling (Hallett and Eaton, 2013), which models the cost‐effectiveness taking into account different levels of adherence to each key decision point along the case pathway. The specific costs and effectiveness of bundles of ‘enabling’ interventions to improve key decision points in the cascade may then be explored. A threshold analysis can then also be used to identify maximum levels of investment to address constraints to the care pathway.

A further emerging approach is to link epidemiological models with bespoke economic or health system models analysing demand and/or supply constraints in a single analytical frame. For example, in one of the Xpert studies cited previously, Lin *et al.* (2011) linked the results of a setting‐specific operational model parameterised with local health facility data on patient flows with a TB transmission model to investigate the influence of capacity constraints on the cost‐effectiveness of Xpert. On the demand side, another paper in this supplement (Terris‐Prestholt *et al.*, 2016) links the results of discrete choice experiments and new technology uptake models with economic evaluations.

### 4.3 Economic evaluations as part of impact evaluations

The preceding approaches may assist researchers to predict cost‐effectiveness prior to new technology introduction. However, substantial implementation research may still be required to understand the full influence of demand and supply factors on the cost‐effectiveness of new technologies in LMICs as technologies are rolled out. Economic evaluations alongside implementation research or after scale‐up remain scarce. Implementation research‐based economic evaluations should be encouraged in order to provide further empirical evidence and deeper understanding of key constraints when assessing the cost‐effectiveness of introducing new technologies globally.

## 5 Conclusion

In conclusion, we argue that economic evaluations of new technologies in LMICs should take a complex intervention perspective and routinely consider demand and supply constraints. If demand and supply constraints are not considered rigorously, local decision makers may consider that the economic evaluations are not relevant to their local context, and scarce resources may be allocated away from more cost‐effective interventions. Proposing this paradigm shift, in terms of defining an ‘intervention’ but considering the substantial empirical challenge, we present a conceptual framework, examples of the framework's application and discuss feasible approaches. We recommend that considering these constraints explicitly be seen as standard ‘best practice’ in economic evaluations in LMICs, even if the analysis remains partial given the substantial analytical and data challenges. We recommend that demand and supply constraints be considered from the earliest stages of the decision process, and that they be incorporated, applied and evaluated throughout the technology adoption process, with at least as much rigour as epidemiological and price heterogeneity.

## Conflict of Interest

The authors have no conflict of interest.
